# COVID-19 and patients with immune-mediated inflammatory diseases undergoing pharmacological treatments: a rapid living systematic review

**DOI:** 10.1590/1516-3180.2020.0421.R2.10092020

**Published:** 2020-12-14

**Authors:** Aline Pereira da Rocha, Álvaro Nagib Atallah, Ana Carolina Pereira Nunes Pinto, César Ramos Rocha-Filho, Keilla Martins Milby, Vinicius Tassoni Civile, Nelson Carvas, Felipe Sebastião de Assis Reis, Laura Jantsch Ferla, Gabriel Sodré Ramalho, Giulia Fernandes Moça Trevisani, Maria Eduarda dos Santos Puga, Virgínia Fernandes Moça Trevisani

**Affiliations:** I MSc. Pharmacist and Doctoral Student, Evidence-Based Health Program, Universidade Federal de São Paulo (UNIFESP), São Paulo (SP), Brazil; and Volunteer Researcher, Cochrane Brazil, São Paulo (SP), Brazil.; II MD, PhD. Nephrologist. Full Professor, Discipline of Emergency and Evidence-Based Medicine, Escola Paulista de Medicina (EPM), Universidade Federal de São Paulo (UNIFESP), São Paulo (SP), Brazil; and Director, Cochrane Brazil, São Paulo (SP), Brazil.; III MSc. Physiotherapist and Doctoral Student, Evidence-Based Health Program, Universidade Federal de São Paulo (UNIFESP), São Paulo (SP), Brazil; Professor, Department of Biological and Health Sciences, Universidade Federal do Amapá, Macapá (AP), Brazil; and Volunteer Researcher, Cochrane Brazil, São Paulo (SP), Brazil.; IV MSc. Biotechnologist and Doctoral Student, Evidence-Based Health Program, Universidade Federal de São Paulo (UNIFESP), São Paulo (SP), Brazil.; V MSc. Nurse and Doctoral Student, Evidence-Based Health Program, Universidade Federal de São Paulo (UNIFESP), São Paulo (SP), Brazil; and Volunteer Researcher, Cochrane Brazil, São Paulo (SP), Brazil.; VI MSc. Physiotherapist and Doctoral Student, Evidence-Based Health Program, Universidade Federal de São Paulo (UNIFESP), São Paulo (SP), Brazil; Assistant Professor, Physiotherapy Course, Universidade Paulista, São Paulo (SP), Brazil; and Volunteer Researcher, Cochrane Brazil, São Paulo (SP), Brazil.; VII Physical Educator and Assistant Professor, Physiotherapy Course, Universidade Ibirapuera, São Paulo (SP), Brazil.; VIII MD, MPS. Manager, Medical Practices, Beneficência Portuguesa de São Paulo, São Paulo (SP), Brazil.; IX Undergraduate Medical Student, Escola Paulista de Medicina (EPM), Universidade Federal de São Paulo (UNIFESP), São Paulo (SP), Brazil.; X Undergraduate Medical Student, Escola Paulista de Medicina (EPM), Universidade Federal de São Paulo (UNIFESP), São Paulo (SP), Brazil.; XI Undergraduate Medical Student, Universidade Santo Amaro, São Paulo (SP), Brazil.; XII Librarian, Evidence-Based Health Program, Universidade Federal de São Paulo (UNIFESP), São Paulo (SP), Brazil.; XIII MD, PhD. Rheumatologist and Professor, Discipline of Emergency and Evidence-Based Medicine, Escola Paulista de Medicina (EPM), Universidade Federal de São Paulo (UNIFESP), São Paulo (SP) Brazil; and Professor, Discipline of Rheumatology, Universidade de Santo Amaro, São Paulo (SP), Brazil.

**Keywords:** Immunosuppressive agents, Antirheumatic agents, Coronavirus infections, Systematic review [publication type], Rheumatic diseases, Immune-mediated inflammatory diseases, Immune-modulating therapies, Evidence-based health

## Abstract

**BACKGROUND:**

Patients with immune-mediated inflammatory diseases (IMID) are at increased risk of infection.

**OBJECTIVE:**

To assess whether patients undergoing pharmacological treatment for IMID present higher risk of worse outcomes when diagnosed with COVID-19.

**DESIGN AND SETTING:**

Rapid systematic review conducted in the medical school of the Federal University of São Paulo (SP), Brazil.

**METHODS:**

We searched CENTRAL, MEDLINE, EMBASE, LILACS, SCOPUS, Web of Science, L·OVE, ClinicalTrials.gov and WHO-ICTRP for studies evaluating patients diagnosed with COVID-19 who were undergoing pharmacological treatment for IMID. Two authors selected studies, extracted data and assessed risk of bias and certainty of evidence, following the Cochrane recommendations.

**RESULTS:**

We identified 1,498 references, from which one cohort study was included. This compared patients with and without rheumatic diseases (RD) who all had been diagnosed with COVID-19. Those with RD seemed to have higher chances of hospitalization and mortality, but no statistical difference was detected between the groups: hospitalization: odds ratio (OR) 1.17; 95% confidence interval (CI) 0.6 to 2.29; mortality rate: OR 1.53; 95% CI 0.33 to 7.11 (very low certainty of evidence). Patients with RD were three times more likely to require admission to intensive care units (ICUs), with invasive mechanical ventilation (IMV), than those without RD: OR 3.72; 95% CI 1.35 to 10.26 (for both outcomes; very low certainty of evidence).

**CONCLUSION:**

Patients undergoing pharmacological treatment for IMID seem to present higher chances of requiring admission to ICUs, with IMV. Additional high-quality studies are needed to analyze the effects of different treatments for IMID.

## INTRODUCTION

In response to the current coronavirus disease (COVID-19) outbreak, many physicians and researchers have been concerned about patients with immune-mediated inflammatory diseases (IMID).^1^^–^^4^ Through immunosuppressive treatment regimens, these patients may be more prone to infections with poor evolution of outcomes.^5^ Although Favalli et al.^3^ showed that the incidence of COVID-19 was quite similar between rheumatic disease patients and individuals in the general population in Lombardy, Italy (0.62% versus 0.66%, respectively), a previous study showed that the most prevalent comorbidity among patients under 40 years old who had been diagnosed with COVID-19 and admitted to ICUs was IMID.^6^

Immunomodulatory therapies have been tested for treating patients with COVID-19. The biological reason for using these drugs is that they mitigate excessive inflammatory responses (cytokine storms), which can cause severe disease and worse prognosis among patients with COVID-19. Therefore, it has also been hypothesized that immunomodulatory therapies have a potential protective effect.^7^ However, neither this therapy nor the protective hypothesis has been proven to be effective.

Although the therapeutic effect of immunomodulatory drugs for treating COVID-19 has been exhaustively explored, the protective effect remains poorly investigated. The protective hypothesis is particularly concerning, since patients under immunomodulatory therapies may neglect preventive measures, including social distancing and the use of personal protective equipment. Analysis on this hypothesis may help decision-makers and healthcare organizations to develop guidelines for management of patients with IMID and identify high-risk individuals during the pandemic.

## OBJECTIVE

To assess whether patients undergoing pharmacological treatment for IMID are at higher risk of worse outcomes when diagnosed with COVID-19.

## METHODS

We used abbreviated systematic review methods, and therefore we did not perform any independent screening of abstracts and did not search the grey literature.^8^ As this was a rapid review, it will be continuously updated (i.e. through monthly searches) and, when any important new evidence is identified, we will analyze the data and update the results.

The protocol for this systematic review was registered on the PROSPERO “International Prospective Register of Systematic Reviews” platform (CRD42020179863).

### Design and setting

The rapid systematic review methodology used here followed the recommendations proposed in the Cochrane Collaboration Handbook. This review was conducted in the medical school of a public university in São Paulo (SP), Brazil.

### Criteria for including reviews

#### Types of studies

We planned to include cohort and case-control studies, and if no better evidence were available, we planned to also consider case series and electronic health records for inclusion.

#### Types of participants

We included participants with IMID who were undergoing pharmacological treatments and who then received a confirmed diagnosis of severe acute respiratory syndrome coronavirus 2 (SARS-CoV-2) infection. Their pharmacological treatment for IMID could include any of the following drugs:

- Immunosuppressants (e.g. azathioprine, mycophenolate or cyclophosphamide);
- Immunomodulators (e.g. glucocorticoids or immunoglobulins);
- Immunobiological agents (e.g. tocilizumab, infliximab, adalimumab, etanercept, certolizumab, rituximab, secukinumab or ustekinumab);
- Synthetic disease-modifying anti-rheumatic drugs (methotrexate, leflunomide, chloroquine or sulfasalazine);
- Targeted synthetic disease-modifying anti-rheumatic drugs (e.g. apremilast, tofacitinib or baricitinib).

#### Types of outcomes

These were our prespecified outcomes:

- Primary outcomes
  – Mortality rate;
  – Length of hospital stay;
  – Adverse events.
- Secondary outcomes
  – Duration of invasive mechanical ventilation;
  – Time to viral clearance;
  – Time to clinical improvement;
  – Length of intensive care unit stay.

### Search strategy

We conducted a systematic search of the literature on July 5, 2020, in the following databases: Medline via PubMed, Embase via Elsevier, Cochrane Library - Cochrane Central Register of Controlled Trials (CENTRAL), BVS Regional Portal (LILACS), Scopus and Web of Science using relevant descriptors and synonyms, with adaptation of the search to the specifications of each database, to identify published, ongoing and unpublished studies. We also searched the following COVID-19 specific databases: Epistemonikos COVID-19 L·OVE platform (https://app.iloveevidence.com/loves/5e6fdb9669c00e4ac072701d); ClinicalTrials.gov (https://ClinicalTrials.gov/ct2/results?cond=COVID-19); and World Health Organization International Clinical Trials Registry Platform (WHO-ICTRP). In addition, we searched the reference lists of the studies included. Studies published in any language since November 2019 were considered for inclusion. The search strategies for each database are presented in Appendix 1.

Two review authors selected the studies for inclusion, extracted data from these studies and assessed the risk of bias in these studies and the certainty of evidence for the outcomes. We planned to assess the possibility of pooling the results from the studies included, into meta-analyses when at least two studies were sufficiently homogeneous in terms of design, participants and outcome measurements. If insufficient information or heterogeneous studies were found, we planned to summarize the results only in a qualitative synthesis.

### Modification of review protocol

In order to improve our rapid systematic review, we decided to perform a broader search strategy than what was presented in the review protocol. Therefore, we also searched for papers published in conference proceedings. Furthermore, to provide a more detailed assessment of the risk of bias, we decided to use Quality Appraisal in Systematic Reviews of Prognosis Studies (QUIPS) rather than the Newcastle-Ottawa Scale.

## RESULTS

### Search results

**Figure 1:**
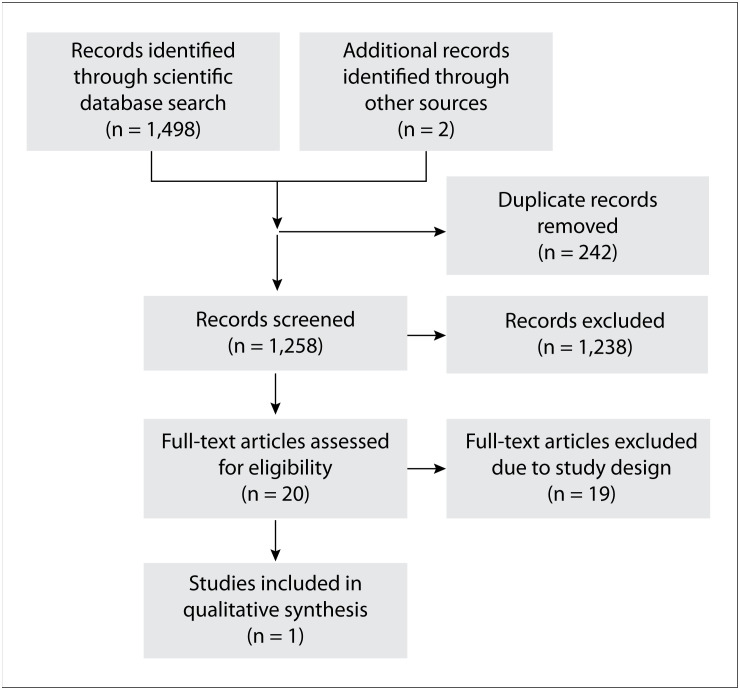
Preferred Reporting Items for Systematic Reviews and Meta-Analyses (PRISMA) flow diagram.

We identified 1,498 reports through our searches in the selected databases and trial registries. After removing duplicates, we screened 1,258 citations, from which we excluded 1,238 reports that did not meet the inclusion criteria. We selected 20 full-text articles^9^^–^^28^ but then we excluded 19 of these.^9^^–^^27^ The reasons for exclusion are shown in the Preferred Reporting Items for Systematic Reviews and Meta-Analyses (PRISMA) flow-chart (Figure 1).

### Characteristics of the included study

We included one retrospective cohort study in our systematic review, which had been conducted in Massachusetts General Hospital and Brigham and Women's Hospital.^28^ This study evaluated 52 patients (mean age 62.5 ± 15.1 years) with SARS-CoV-2 infection and the following rheumatic diseases: rheumatoid arthritis (19 patients), systemic lupus erythematosus (10), polymyalgia rheumatica (7), seronegative spondyloarthritis (7), myositis (3), giant cell arteritis (1), sarcoidosis (1), small vessel vasculitis (2), juvenile idiopathic arthritis (1) and Kikuchi's disease (1) and a control group of 104 participants (mean age 63.1 ± 14.9 years) without rheumatic diseases. In both groups, 69% of the participants were female. The participants in the rheumatic disease group (RDG) had the following comorbidities: hypertension (34 patients), diabetes (13), coronary artery disease (12), heart failure (4) and pulmonary disease (21); while the participants in the control group had hypertension (50 individuals), diabetes (29), coronary artery disease (10), heart failure (11) and pulmonary disease (28). The participants with rheumatic disease were under pharmacological treatment, including: hydroxychloroquine (9 patients), hydroxychloroquine monotherapy (5), tumor necrosis factor (TNF) inhibitor (7), interleukin 6 (IL-6) receptor inhibitor (1), belimumab (2), rituximab (3), interleukin 12/interleukin 23 (IL-12/IL-23) inhibitor (2), abatacept (1), tofacitinib (3), methotrexate (9), leflunomide (4), mycophenolate mofetil (3) and prednisone (5). The patients with rheumatic disease and SARS-CoV-2 infection were compared with the patients with SARS-CoV-2 who did not have rheumatic diseases (control group, CG), regarding comorbidities, age, race and gender.

### Excluded studies

We read 20 full-text articles to assess the possibility of inclusion. We excluded 4 case series and 15 case-report studies,^9^^–^^27^ because these study designs were not appropriate for assessing prognosis questions.

### Risk of bias in the included study

**Figure 2:**
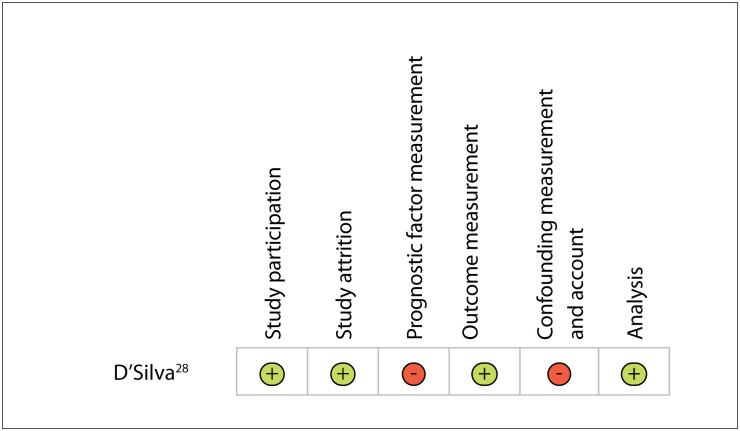
Risk of bias in the study included.

We assessed the risk of bias in the retrospective cohort study using Quality Appraisal in Systematic Reviews of Prognosis Studies (QUIPS).^28^^,^^29^ The study received two negative assessments, in relation to prognostic factor measurement and to confounding measurement and account criteria, because of multiple drug therapy used in the RDG (without adjustment for the confounders, for instance). We have summarized the risk of bias assessments in Figure 2.

### Certainty of evidence

We rated the certainty of the evidence using the Grading of Recommendations Assessment, Development and Evaluation (GRADE) approach.^30^ We found very low certainty of evidence for all the reported outcomes. We downgraded by one level in situations of study limitation (risk of bias), by one level in situations of indirectness (important differences in the severity of the different rheumatic diseases) and by one level in situations of imprecision of effect estimation.

### Outcome results

Among the outcomes of interest, only hospitalization rate, length of hospital stay, ICU admission rate, need for invasive mechanical ventilation (IMV), duration of IMV support and mortality were evaluated in the study included in this review. The following outcomes were not reported: length of ICU stay, adverse events, time to viral clearance and time to clinical improvement. The results and certainty of evidence for each outcome measurement and the effect size (odds ratio and mean difference) are shown in the “Summary of findings” table (Table 1).

**Table 1:** Summary of findings

**Rheumatic patients undergoing treatments with immunosuppressants, immunobiological agents, synthetic DMARDs or targeted synthetic DMARDs, compared with participants without rheumatic diseases; both groups diagnosed with COVID-19**
**Patient or population:** Rheumatic patients using DMARDs, immunobiological agents, immunosuppressants or corticosteroid who were then diagnosed with COVID-19.
**Comparison:** Participants without rheumatic diseases and not undergoing no drug treatment, who had been diagnosed with COVID-19.
**Setting:** Tertiary-level care and community hospitals; and primary and specialty outpatient centers.

Outcomes	Relative effect (95% CI)	No. of participants (no. of studies)	Certainty of the evidence (GRADE)
Hospitalization	**OR 1.1** (0.6 to 2.29)	156 (1 observational study)	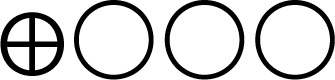 VERY LOW abc
Length of hospital stay	Mean difference in length of hospital stay between the groups was 1.3 (-4.85 to 7.34) days	65 (1 observational study)	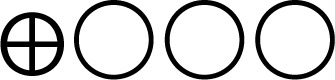 VERY LOW abc
ICU admission	**OR 3.72** (1.35 to 10.26)	156 (1 observational study)	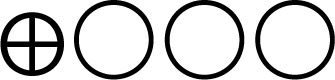 VERY LOW abc
Mechanical ventilation	**OR 3.72** (1.35 to 10.26)	156 (1 observational study)	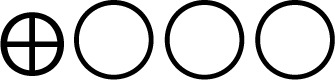 VERY LOW abc
Mortality	**OR 1.53** (0.33 to 7.11)	156 (1 observational study)	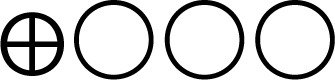 VERY LOW abc

DMARDs = disease-modifying antirheumatic drugs; CI = confidence interval; OR = odds ratio; ICU: intensive care unit; length of hospital stay is expressed as the mean number of days (with standard deviation).
**GRADE Working Group grades of evidence**
**High certainty:** Further research is very unlikely to change our confidence in the estimate of effect.
**Moderate certainty:** Further research is likely to have an important impact on our confidence in the estimate of effect and may change the estimate.
**Low certainty:** Further research is very likely to have an important impact on our confidence in the estimate of effect and is likely to change the estimate
**Very low certainty:** We are very uncertain about the estimate.

**Explanations**

a Downgraded by one level due to serious study limitations (risk of bias).

b Downgraded by one level due to serious indirectness. Important differences in the severity of different rheumatic diseases.

c Downgraded by one level due to serious imprecision. The 95% CI crossed the line of no effect and was also a wide interval around the estimate of the effect. Therefore, it was clinically irrelevant.

#### Hospitalization

Although the patients with RD seemed to have a higher chance of hospitalization, we could not detect any statistically significant difference between the groups. In the RDG, 23 patients were hospitalized versus 42 patients in the CG: odds ratio (OR) = 1.17; 95% confidence interval (CI) 0.6 to 2.29. The mean difference between the groups regarding the length of hospital stay was 1.30 days (95% CI 4.85 to 7.45).

#### Intensive care unit (ICU) admission

The RDG presented three times more chance of being admitted to an ICU than participants in the CG (OR 3.72; 95% CI 1.35 to 10.26).

#### Invasive mechanical ventilation (IMV)

The number of patients who received IMV was statistically greater in the RDG (11 patients) than in the CG (7 patients) (OR 3.72; 95% CI 1.35 to 10.26). The mean difference between the two groups regarding mechanical ventilation was 3.14 days (95% CI 1.29 to 7.63).

#### Mortality

Although the patients in the RD group seemed to have a higher chance of mortality, we could not detect any statistically significant difference between the groups (OR 1.53; 95% CI 0.33 to 7.11).

## DISCUSSION

This was the first systematic review to evaluate whether patients with IMID undergoing pharmacological treatment with immunosuppressants, immunobiological agents, synthetic disease-modifying antirheumatic drugs (DMARDs) or targeted synthetic DMARDs have better or worse outcomes when infected by SARS-CoV-2. A single retrospective study^28^ provided very low certainty of evidence that patients with IMID who were undergoing long-term pharmacological treatments seemed to have higher chances of hospitalization and mortality. However, comparison with patients without IMID and who were not undergoing treatments with immunosuppressants, immunobiological agents, synthetic DMARDs or targeted synthetic DMARDs did not show any statistically significant difference in these chances. There was also very low certainty of evidence from the same study that the chances of being admitted to an ICU and of needing IMV were higher in the RDG than among patients without IMID who were not receiving these long-term pharmacological treatments.

Several limitations of the study that was included in the present review need to be highlighted. Firstly, the RDG was composed of participants with several types of IMID and with different severities of disease. Secondly, the participants with IMID were under several drug treatments and no specific analyses taking into account the type of drug were conducted. Lastly, no information on drug dose and duration of drug treatment was provided. Therefore, we were unable to directly investigate the influence of each class of drugs on the course of COVID-19 in patients with IMID who were undergoing specific pharmacological treatments.

Our results are in line with those from a previous study that included 1,591 consecutive patients referred for ICU admission. That study showed that IMID was the most prevalent comorbidity in patients with laboratory confirmation as positive for SARS-CoV-2 who were admitted to ICUs.^6^ The current systematic review also found one retrospective cohort study^28^ suggesting that patients with IMID may be more likely to have worse evolution when infected by SARS-CoV-2. Although the latter study had a large sample, it was a retrospective case series and therefore it was excluded from the present review.

We took efforts to rapidly identify all the available evidence, through a broad and sensitive search. In spite of this, the studies identified were not appropriate for answering the clinical question of this review. We identified 19 studies (4 case series and 15 case reports) that discussed clinical and laboratory findings from patients with IMID, but several methodological limitations of the present review need to be taken into account. Firstly, the studies identified did not have control groups and we were unable to examine whether participants who were not under pharmacological treatment for IMID had better or worse outcomes. Secondly, we did not find any studies that evaluated potential adverse effects of long-term use of these drugs after the presence of SARS-CoV-2 infection had been diagnosed, or the time to viral clearance, time to clinical improvement or length of ICU stay. Lastly, none of the studies identified had been prospectively planned for evaluation of the question of this review.

Given that the current pandemic scenario has exposed shortages of professionals and resources, along with limitations to evidence-based clinical protocols, the outcomes of critical clinical importance would be those relating to the duration of usage of limited resources, such as the time taken to achieve clinical improvement, time to viral clearance and length of ICU stay. We are aware that the difficulties involved in designing and conducting studies during these times of pandemic have contributed to the dearth of high-quality studies. We are also conscious that the heterogeneous patient groups, multiple classes of drugs and multiple methodologies among the various studies conducted have added complications to standardized data extraction procedures, such as those required for systematic reviews and meta-analyses.

We believe that there is a great need for prospective cohorts to be conducted in the future with the aim of examining representative samples of patients with IMID undergoing pharmacological treatments who are then diagnosed with COVID-19. Adjustments will need to be made for confounding variables such as in relation to use of multiple drugs, administration route, disease severity, comorbidities and age. Through such studies, the level of confidence in the effect estimates can be improved.

The current evidence was assessed in the present review through methodological appraisal. Although this has provided scientifically rigorous data to inform further studies, the results reported here should be interpreted cautiously in analyses for decision-making processes.

## CONCLUSION

To date, based on the results from a single retrospective cohort study, no protective effect from the drugs used for treating IMID, regarding the clinical course of COVID-19, has been demonstrated. On the contrary, patients with IMID seem to have higher chances of being admitted to ICUs and of requiring IMV. Furthermore, additional high-quality studies are needed in order to analyze the effects of different treatments for IMID, while considering the characteristics of the disease and the treatment administered on an individualized basis, among patients who also present infection with COVID-19.

## APPENDIX 1. Search strategies.

### MEDLINE via PubMed.

"COVID-19" [Supplementary Concept OR (COVID 19) OR (COVID-19) OR (2019-nCoV) OR (nCoV) OR (Covid19) OR (SARS-CoV) OR (SARSCov2 or ncov*) OR (SARSCov2) OR (2019 coronavirus*) OR (2019 corona virus*) OR (Coronavirus (COVID-19)) OR (2019 novel coronavirus disease) OR (COVID-19 pandemic) OR (COVID-19 virus infection) OR (coronavirus disease-19) OR (2019 novel coronavirus infection) OR (2019-nCoV infection) OR (coronavirus disease 2019) OR (2019-nCoV disease) OR (COVID-19 virus disease) OR "severe acute respiratory syndrome coronavirus 2" [Supplementary Concept] OR (Wuhan coronavirus) OR (Wuhan seafood market pneumonia virus) OR (COVID19 virus) OR (COVID-19 virus) OR (coronavirus disease 2019 virus) OR (SARS-CoV-2) OR (SARS2) OR (2019-nCoV) OR (2019 novel coronavirus)

"Interleukin-6"[Mesh] OR interleukin 6 OR "IL 6" OR IL-6 OR IL6 OR "tocilizumab" [Supplementary Concept] OR Tocilizum* OR altizumab OR actemra OR RHPM-1 OR RG-1569 OR R-1569 OR MSB11456 OR MSB-11456 OR (monoclonal antibody, MRA) OR (RO-4877533) OR roactemra OR anti-IL-6 OR anti-interleukin-6 OR "siltuximab" [Supplementary Concept] OR CLLB8 OR (cClB8 monoclonal antibody) OR Sylvant OR CNTO-328 OR (CNTO 328 monoclonal antibody) OR (monoclonal antibody CNTO328) OR "sarilumab" [Supplementary Concept] OR SAR-153191 OR SAR153191 OR Kevzara OR REGN-88 OR REGN88 OR "olokizumab" [Supplementary Concept] OR CDP-6038 OR CDP6038 OR elsilimomab OR BMS945429 OR ALD518 OR "sirukumab" [Supplementary Concept] OR (CNTO 136) OR CNTO-136 OR CPSI-2364 OR ALX-0061 OR "clazakizumab" [Supplementary Concept] OR ALD-518 OR ALD518 OR BMS-945429 OR "sarilumab" [Supplementary Concept] OR SAR-153191 OR SAR153191 OR Kevzara OR REGN-88 OR REGN88 OR "sirukumab" [Supplementary Concept] OR ARGX-109 OR FE301 OR FM101 OR "Tumor Necrosis Factor-alpha"[Mesh] OR TNF OR TNF-alpha OR TNF-α OR Anti-TNF OR "Infliximab"[Mesh] OR (Monoclonal Antibody cA2) OR (MAb cA2) OR Infliximab-abda OR Renflexis OR Infliximab-dyyb OR Inflectra OR Remicade OR "Etanercept"[Mesh] OR (TNFR-Fc Fusion Protein) OR (TNR 001) OR (TNT Receptor Fusion Protein) OR TNTR-Fc OR TNR-001 OR TNR001 OR Etanercept-szzs OR (TNF Receptor Type II-IgG Fusion Protein) OR (TNF Receptor Type II IgG Fusion Protein) OR Erelzi OR (Recombinant Human Dimeric TNF Receptor Type II-IgG Fusion Protein) OR (Recombinant Human Dimeric TNF Receptor Type II IgG Fusion Protein) OR Enbrel OR "Certolizumab Pegol"[Mesh] OR Certolizumab OR Cimzia OR CDP870 OR (CDP 870) OR "golimumab" [Supplementary Concept] OR CNTO-148 OR (CNTO 148) OR Simponi OR "Adalimumab"[Mesh] OR Humira OR Adalimumab-adbm OR Amjevita OR Adalimumab-atto OR Cyltezo OR (D2E7 Antibody) OR "Interleukin-1"[Mesh] OR IL-1 OR IL-1RA OR "IL 1" OR "canakinumab" [Supplementary Concept] OR ilaris OR ACZ-885 OR ACZ885 OR anti-IL-1 OR "rilonacept" [Supplementary Concept] OR ACZ885 OR anakinra OR "Interleukin-5"[Mesh] OR Anti-IL-5 OR "mepolizumab" [Supplementary Concept] OR Bosatria OR SB-240563 OR SB240563 OR Nucala OR "Interleukin-12"[Mesh] OR IL-12 OR "Ustekinumab"[Mesh] OR Stelara OR (CNTO 1275) OR CNTO-1275 OR "Interleukin-23"[Mesh] OR IL-23 OR "IL 23" OR "briakinumab" [Supplementary Concept] OR A-796874.0 OR BSF-415977 OR (BSF 415977) OR WAY-165772 OR LU-415977 OR (LU 415977) OR J-695 OR J695 OR ABT-874 OR (ABT-874 antibody, human) OR Anti-C5 OR "eculizumab" [Supplementary Concept] OR Alexion OR Soliris OR 5G1.1 OT (H5G1.1VHC+H5G1.1VLC) OR H5G1.1 OR H5G1-1 OR H5G11 OR "Abatacept"[Mesh] OR LEA29Y OR BMS224818 OR BMS-224818 OR (BMS 224818) OR Belatacept OR (BMS 188667) OR (BMS-188667) OR CTLA-4-Ig OR (Cytotoxic T Lymphocyte-Associated Antigen 4-Immunoglobulin) OR (Cytotoxic T Lymphocyte Associated Antigen 4 Immunoglobulin) OR CTLA4-Ig OR (CTLA4-Ig Immunoconjugate) OR (CTLA4 Ig Immunoconjugate) OR (Immunoconjugate, CTLA4-Ig) OR CTLA4-Fc OR Nulojix OR "Rituximab"[Mesh] OR (CD20 Antibody) OR (Rituximab CD20 Antibody) OR Mabthera OR (IDEC-C2B8 Antibody) OR (IDEC C2B8 Antibody) OR (IDEC-C2B8) OR (IDEC C2B8) OR GP2013 OR Rituxan OR "Antigens, CD20"[Mesh] OR (CD20 Antigen) OR (CD20 Antigens) OR "belimumab" [Supplementary Concept] OR (BEL-114333) OR BEL114333 OR HGS-1006 OR HGS1006 OR LymphoStat-B OR GSK-1550188 OR GSK1550188 OR Benlysta OR "secukinumab" [Supplementary Concept] OR "Interleukin-17"[Mesh] OR IL-17A OR IL-17 OR "IL 17" OR "ixekizumab" [Supplementary Concept] OR "brodalumab" [Supplementary Concept] OR "guselkumab" [Supplementary Concept] OR "tildrakizumab" [Supplementary Concept] OR "risankizumab" [Supplementary Concept] OR "apremilast" [Supplementary Concept] OR Otezla OR (CC 10004) OR CC10004 OR CC-10004 OR "tofacitinib" [Supplementary Concept] OR tasocitinib OR (tofacitinib citrate) OR Xeljanz OR (CP 690,550) OR CP690550 OR CP-690550 OR (CP 690550) OR CP-690,550 OR "baricitinib" [Supplementary Concept] OR LY3009104 OR Olumiant OR INCB028050 OR "Azathioprine"[Mesh] OR Azothioprine OR Imurel OR Imuran OR Immuran OR "Mycophenolic Acid"[Mesh] OR (Mycophenolate Mofetil) OR Cellcept OR (Mycophenolate Sodium) OR Myfortic OR (RS 61443) OR (RS-61443) OR RS61443 OR "Cyclophosphamide"[Mesh] OR Sendoxan OR B-518 OR (B 518) OR B518 OR Cytophosphane OR (Cyclophosphamide Monohydrate) OR Cytophosphan OR Cytoxan OR Endoxan OR Neosar OR NSC-26271 OR (NSC 26271) OR NSC26271 OR Procytox OR Cyclophosphane OR "Cyclosporine"[Mesh] OR Ciclosporin OR Cyclosporin OR Neoral OR (Sandimmun Neoral) OR (CyA-NOF) OR (CyA NOF) OR Sandimmune OR Sandimmun OR (CsA-Neoral) OR (CsA Neoral) OR CsANeoral OR (OL 27-400) OR (OL 27 400) OR (OL 27400) OR "Tacrolimus"[Mesh] OR Prograft OR FR-900506 OR (FR 900506) OR FR900506 OR (Anhydrous Tacrolimus) OR FK-506 OR (FK 506) OR FK506 OR "Hydroxychloroquine"[Mesh] OR (Hydroxychloroquine) OR Oxychlorochin OR Oxychloroquine OR Hydroxychlorochin OR Plaquenil OR Hidroxicloroquina OR Hydroxychloroquinum OR Oxichlorochine OR Oxicloroquine OR "Chloroquine"[Mesh] OR Chlorochin OR Cloroquina OR Cloroquine OR Chloroquine OR "Antimalarials"[Mesh] OR Antimalarials OR Anti-Malarials OR (Anti Malarials) OR Hydroquin OR Axemal OR Dolquine OR Quensyl OR Quinoric OR "Sulfasalazine"[Mesh] OR Salicylazosulfapyridine OR (Pyralin EN) OR Azulfadine OR Azulfidine OR Asulfidine OR (Colo-Pleon) OR (Colo Pleon) OR Pleon OR Ulcol OR Sulfasalazin OR Ucine OR Salazopyrin OR (ratio-Sulfasalazine) OR (ratio Sulfasalazine) OR "Methotrexate"[Mesh] OR Amethopterin OR Mexate OR "Leflunomide"[Mesh] OR (HWA 486) OR HWA-486 OR HWA486 OR SU101 OR Arava OR "Dapsone"[Mesh] OR DADPS OR Sulfonyldianiline OR Diaminodiphenylsulfone OR Diaphenylsulfone OR (4,4'-Diaminophenyl Sulfone) OR (4,4' Diaminophenyl Sulfone) OR Sulfona OR (Dapson-Fatol) OR Disulone OR Avlosulfone OR (Dapsoderm-X) OR "Glucocorticoids"[Mesh] OR Glucocorticoid OR "Immunoglobulins"[Mesh] OR Immunoglobulin OR Globulins

**Publication date from 2019/11/01**

#1 AND #2 AND #3

### EMBASE

#1 ‘covid 19’/exp OR (COVID 19) OR (COVID-19) OR (2019-nCoV) OR (nCoV) OR (Covid19) OR (SARS-CoV) OR (SARSCov2 or ncov*) OR (SARSCov2) OR (2019 coronavirus*) OR (2019 corona virus*) OR (Coronavirus (COVID-19)) OR (2019 novel coronavirus disease) OR (COVID-19 pandemic) OR (COVID-19 virus infection) OR (coronavirus disease-19) OR (2019 novel coronavirus infection) OR (2019-nCoV infection) OR (coronavirus disease 2019) OR (2019-nCoV disease) OR (COVID-19 virus disease) OR (severe acute respiratory syndrome coronavirus 2) OR (Wuhan coronavirus) OR (Wuhan seafood market pneumonia virus) OR (COVID19 virus) OR (COVID-19 virus) OR (coronavirus disease 2019 virus) OR (SARS-CoV-2) OR (SARS2) OR (2019-nCoV) OR (2019 novel coronavirus)

#2 ‘interleukin 6’/exp OR ‘interleukin 6’ OR ‘IL 6’ OR interleukin-6 OR ‘tocilizumab’/exp OR tocilizumab OR actemra OR atlizumab OR lusinex OR ‘r 1569’ OR r1569 OR roactemra OR ‘sarilumab’/exp OR sarilumab OR kevzara OR ‘regn 88’ OR regn88 OR ‘sar 153191’ OR sar153191 OR ‘tumor necrosis factor inhibitor’/exp OR ‘tumor necrosis factor’ OR ‘TNF alpha’ OR TNF-alpha OR TNF OR ‘tumour necrosis factor’ OR ‘infliximab’/exp OR ‘abp 710’ OR abp710 OR avakine OR flixabi OR ‘gp 1111’ OR gp1111 OR inflectra OR infliximab-abda OR infliximab-dyyb OR infliximab-qbtx OR ixifi OR ‘pf 06438179’ OR ‘pf 6438179’ OR pf06438179 OR pf6438179 OR remicade OR remsima OR renflexis OR revellex OR ‘ta 650’ OR ta650 OR zessly OR ‘etanercept’/exp OR etanercept OR avent OR benepali OR embrel OR enbrel OR enerceptan OR ‘enia 11’ OR enia11 OR erelzi OR etanercept-szzs OR etanercept-ykro OR eticovo OR ‘gp 2015’ OR gp2015 OR infinitam OR lifmior OR opinercept OR ‘tnr 001’ OR tnr001 OR tunex OR ‘ylb 113’ OR ylb113 OR ‘certolizumab’/exp OR certolizumab OR ‘golimumab’/exp OR ‘cnto 148’ OR cnto148 OR simponi OR ‘golimumab’/exp OR golimumab OR ‘cnto 148’ OR cnto148 OR simponi OR ‘adalimumab’/exp OR ‘abp 501’ OR abp501 OR ‘abt d2e7’ OR abtd2e7 OR adalimumab-adaz OR adalimumab-adbm OR adalimumab-atto OR adalimumab-bwwd OR adaly OR amgevita OR amjevita OR ‘avt 02’ OR avt02 OR ‘bat 1406’ OR bat1406 OR ‘bax 2923’ OR ‘bax 923’ OR bax2923 OR bax923 OR ‘bi 695501’ OR bi695501 OR ‘chs 1420’ OR chs1420 OR ‘ct p17’ OR ctp17 OR cyltezo OR ‘da 3113’ OR da3113 OR ‘dmb 3113’ OR dmb3113 OR exemptia OR ‘fkb 327’ OR fkb327 OR fyzoclad OR ‘gp 2017’ OR gp2017 OR hadlima OR halimatoz OR hefiya OR ‘hlx 03’ OR hlx03 OR hulio OR humira OR hyrimoz OR ‘ibi 303’ OR ibi303 OR idacio OR imraldi OR kromeya OR ‘lu 200134’ OR lu200134 OR ‘m 923’ OR m923 OR mabura OR ‘monoclonal antibody D2E7’ OR ‘msb 11022’ OR msb11022 OR ‘ons 3010’ OR ons3010 OR ‘pf 06410293’ OR ‘pf 6410293’ OR pf06410293 OR pf6410293 OR raheara OR ‘sb 5’ OR sb5 OR solymbic OR trudexa OR ‘zrc 3197’ OR zrc3197 OR ‘interleukin 1’/exp OR ‘interleukin 1’ OR ‘IL 1’ OR IL-1 OR ‘interleukin I’ OR interleukin-1 OR ‘canakinumab’/exp OR ‘acz 885’ OR acz885 OR ilaris OR ‘rilonacept’/exp OR rilonacept OR arcalyst OR ‘anakinra’/exp OR anakira OR kineret OR ‘interleukin 5’/exp OR ‘interleukin 5’ OR ‘il 5’ OR interleukin-5 OR IL-5 OR ‘mepolizumab’/exp OR mepolizumab OR bosatria OR nucala OR ‘sb 240563’ OR sb-240563 OR sb240563 OR ‘interleukin 12’/exp OR ‘interleukin 12’ OR ‘IL 12’ OR il-12 OR interleukin-12 OR ‘interleukin 23’/exp OR ‘interleukin 23’ OR ‘IL 23’ OR interleukin-23 OR ‘ustekinumab’/exp OR ustekinumab OR ‘cnto 1275’ OR cnto1275 OR stelara OR ‘eculizumab’/exp OR eculizumab OR ‘monoclonal antibody 5G1.1’ OR soliris OR ‘abatacept’/exp OR abatacept OR ‘bms 188667’ OR bms188667 OR ‘CTLA4 Ig’ OR ‘CTLA4 immunoglobulin’ OR ‘CTLA4 immunoglobulin G’ OR CTLA4Ig OR orencia OR ‘rituximab’/exp OR rituximab OR ‘abp 798’ OR ‘abp798’ OR blitzima OR ‘ct p10’ OR ctp10 OR ‘gp 2013’ OR gp2013 OR ‘hlx 01’ OR hlx01 OR ‘idec 102’ OR ‘idec c2b8’ OR idec102 OR idecc2b8 OR mabthera OR ‘mk 8808’ OR mk8808 OR ‘monoclonal antibody idec c2b8’ OR ‘pf 05280586’ OR ‘pf 5280586’ OR pf05280586 OR pf5280586 OR ‘r 105’ OR r105 OR reditux OR ‘rg 105’ OR rg105 OR ritemvia OR ritumax OR rituxan OR rituximab-abbs OR rituximab-pvvr OR rituxin OR rituzena OR rixathon OR riximyo OR ‘ro 452294’ OR ro452294 OR ruxience OR truxima OR tuxella OR ‘belimumab’/exp OR belimumab OR benlysta OR ‘lymphostat B’ OR ‘interleukin 17’/exp OR ‘interleukin 17’ OR ‘il 17A’ OR IL-17 OR ‘interleukin 17A’ OR interleukin-17 OR ‘secukinumab’/exp OR secukinumab OR ‘ain 457’ OR ain457 OR cosentyx OR ‘ixekizumab’/exp OR ixekizumab OR ‘ly 2439821’ OR ly2439821 OR taltz OR ‘brodalumab’/exp OR brodalumab OR ‘amg 827’ OR amg827 OR kyntheum OR siliq OR ‘guselkumab’/exp OR guselkumab OR ‘cnto 1959’ OR cnto1959 OR tremfya OR ‘tildrakizumab’/exp OR tildrakizumab OR ilumetri OR ilumya OR ‘mk 3222’ OR ‘mk3222’ OR ‘sch 900222’ OR sch900222 OR ‘sunpg 1622’ OR ‘sunpg 1623’ OR sunpg1622 OR sunpg1623 OR ‘tildrakizumab asmn’ OR tildrakizumab-asmn OR ‘risankizumab’/exp OR risankizumab OR ‘abbv 066’ OR abbv066 OR ‘bi 655066’ OR bi655066 OR ‘risankizumab rzaa’ OR risankizumab-rzaa OR skyrizi OR ‘apremilast’/exp OR apremilast OR ‘cc 10004’ OR cc10004 OR otezla OR ‘tofacitinib’/exp OR tofacitinib OR ‘cp 690 550’ OR ‘cp 690, 550’ OR ‘cp 690550’ OR ‘cp 690550-10’ OR ‘cp690 550’ OR ‘cp690, 550’ OR cp690550 OR cp690550-10 OR tasocitinib OR ‘tofacitinib citrate’ OR xeljanz OR ‘xeljanz xr’ OR ‘baricitinib’/exp OR baricitinib OR ‘incb 028050’ OR ‘incb 28050’ OR incb028050 OR incb28050 OR ‘ly 3009104’ OR ly3009104 OR olumiant OR ‘azathioprine’/exp OR azathioprine OR arathioprin OR arathioprine OR ‘aza-q’ OR azafalk OR azahexal OR azamedac OR azamun OR azamune OR azanin OR azapin OR azapress OR azaprine OR azarex OR azasan OR azathiodura OR azathiopine OR azathioprim OR azathioprin OR azathiopurine OR azathropsin OR azatioprina OR azatox OR azatrilem OR azopi OR azoran OR azothioprin OR azothioprine OR ‘bw 57 322’ OR ‘bw 57-322’ OR ‘w 57322’ OR bw57-322 OR bw57322 OR colinsan OR immuran OR immurel OR immuthera OR imunen OR imuprin OR imuran OR imurane OR imurek OR imurel OR imuren OR ‘nsc 39084’ OR nsc39084 OR thioazeprine OR thioprine OR transimune OR zytrim OR ‘mycophenolate mofetil’/exp OR ‘mycophenolate mofetil’ OR ‘cell cept’ OR cellcept OR cellmune OR cellsept OR munoloc OR myclausen OR ‘mycophenolic acid 2 morpholinoethyl ester’ OR ‘mycophenolic acid mofetil’ OR myfenax OR ‘rs 61443’ OR ‘rs 61443 190’ OR rs61443 OR ‘rs61443 190’ OR ‘cyclophosphamide’/exp OR cychophosphamide OR alkyroxan OR ‘b 518’ OR ‘b 518 asta’ OR b518 OR ‘b518 asta’ OR carloxan OR ciclofosfamida OR ciclolen OR cicloxal OR clafen OR cyclo-cell OR cycloblastin OR cycloblastine OR ‘cyclofos amide’ OR cyclofosfamid OR cyclofosfamide OR cyclophar OR cyclophosphamid OR ‘cyclophosphamide isopac’ OR cyclophosphamides OR cyclophosphan OR cyclophosphane OR cyclostin OR cycloxan OR cyphos OR cytophosphan OR cytophosphane OR cytoxan OR ‘endocyclo phosphate’ OR endoxan OR ‘endoxan-asta’ OR endoxana OR endoxon-asta OR enduxan OR genoxal OR ledoxan OR ledoxina OR mitoxan OR neosan OR neosar OR noristan OR ‘nsc 26271’ OR nsc2671 OR procytox OR procytoxide OR semdoxan OR sendoxan OR syklofosfamid OR ‘cyclosporine’/exp OR cyclosporine OR ‘adi 628’ OR adi628 OR cequa OR ‘cgc 1072’ OR cgc1072 OR ciclomulsion OR cicloral OR ciclosporin OR ciclosporine OR cipol OR cipol-n OR consupren OR cyclasol OR cyclokat OR cyclosporin OR ‘de 076’ OR de076 OR deximune OR equoral OR gengraf OR ikervis OR iminoral OR implanta OR imusporin OR ‘lx 201’ OR lx201 OR ‘mc2 03’ OR mc203 OR ‘mtd 202’ OR mtd202 OR neoral OR neoral-sandimmun OR ‘neuro-stat drug’ OR ‘neurostat drug’ OR ‘nm 0133’ OR ‘nm 133’ OR nm0133 OR nm133 OR ‘nova 22007’ OR nova22007 OR ‘ol 27400’ OR ol27400 OR ‘olo 400’ OR olo500 OR ‘opph 088’ OR opph088 OR opsisporin OR ‘otx 101’ OR otx101 OR ‘p 3072’ OR p3072 OR padciclo OR papilock OR pulminiq OR restasis OR restaysis OR sanciclo OR sandimmun OR sandimmune OR sandimun OR sandimune OR ‘sang 35’ OR sang35 OR sangcya OR ‘sp 14019’ OR sp14019 OR ‘sti 0529’ OR sti0529 OR ‘t 1580’ OR t1580 OR vekacia OR verkazia OR ‘tacrolimus’/exp OR tacrolimus OR advagraf OR astagraf OR envarsus OR ‘fk 506’ OR fk-506 OR fk506 OR ‘fr 900506’ OR fr900506 OR fujimycin OR hecoria OR modigraf OR ‘mustopic oint’ OR prograf OR prograft OR protopic OR protopy OR tacforius OR ‘tacrolimus hydrate’ OR tsukubaenolide

#3 ‘hydroxychloroquine’/exp OR hydroxychloroquine OR ‘chloroquinol’/exp OR chloroquinol OR ‘ercoquin’/exp OR ercoquin OR ‘hydrochloroquine’/exp OR hydrochloroquine OR ‘hydrocloroquine’/exp OR hydrocloroquine OR ‘oxychloroquine’/exp OR oxychloroquine OR ‘quensyl’/exp OR quensyl OR ‘sn 8137’/exp OR ‘sn 8137’ OR oxychlorochin OR hydroxychlorochin OR plaquenil OR hidroxicloroquina OR hydroxychloroquinum OR oxichloroquine OR ‘chloroquine’/exp OR chloroquine OR a-cq OR amokin OR amokine OR anoclor OR aralan OR aralen OR aralene OR arechin OR arechine OR arequine OR arthrochin OR arthrochine OR arthroquine OR artrichin OR artrichine OR artriquine OR avloclor OR avoclor OR bemaphata OR bemaphate OR bemasulph OR bipiquin OR cadiquin OR chemochin OR chemochine OR chingamine OR chingaminum OR chloraquine OR chlorochin OR chlorochine OR chlorofoz OR chloroquin OR ‘chloroquin phosphate’ OR chloroquinesulphate OR ‘chloroquini diphosphas’ OR ‘chloroquinum diphosphoricum’ OR chlorquin OR chlorquine OR choloquine OR ‘choroquine sulfate’ OR ‘choroquine sulphate’ OR cidanchin OR ‘clo-kit junior’ OR clorichina OR clorichine OR cloriquine OR clorochina OR delagil OR delagyl OR dichinalex OR diclokin OR diquinalex OR diroquine OR emquin OR genocin OR gontochin OR gontochine OR gontoquine OR heliopar OR imagon OR iroquine OR klorokin OR klorokine OR klorokinfosfat OR lagaquin OR malaquin OR malarex OR malarivon OR malaviron OR maliaquine OR maquine OR mesylith OR mexaquin OR mirquin OR nivachine OR nivaquin OR nivaquine OR ‘p roquine’ OR quinachlor OR quingamine OR repal OR resochen OR resochene OR resochin OR ‘resochin junior’ OR resochina OR resochine OR resochinon OR resoquina OR resoquine OR reumachlor OR roquine OR ‘rp 3377’ OR rp3377 OR sanoquin OR sanoquine OR silbesan OR siragan OR sirajan OR ‘sn 7618’ OR sn7618 OR solprina OR solprine OR tresochin OR tresochine OR tresoquine OR trochin OR trochine OR troquine OR ‘w 7618’ OR w7618 OR ‘win 244’ OR win244 OR ‘antimalarial agent’/exp OR ‘antimalarial agent’ OR ‘anti malaria drug’/exp OR ‘anti malaria drug’ OR ‘antimalaria agent’/exp OR ‘antimalaria agent’ OR ‘antimalaria drug’/exp OR ‘antimalaria drug’ OR ‘antimalaria drug, synthetic’/exp OR ‘antimalarial’/exp OR antimalarial OR ‘antimalarial drug’/exp OR ‘antimalarial drug’ OR ‘antimalarials’/exp OR antimalarials OR ‘antipaludean agent’/exp OR ‘antipaludean agent’ OR ‘antiplasmodic agent’/exp OR ‘antiplasmodic agent’ OR ‘synthetic antimalaria agent’/exp OR ‘synthetic antimalaria agent’ OR ‘salazosulfapyridine’/exp OR salazosulfapyridine OR ‘azlufidine en-tabs’ OR azopyrin OR azopyrine OR azosulfidine OR azulfide OR azulfidina OR azulfidine OR ‘azulfidine EN tabs’ OR ‘azulfidine en-tabs’ OR ‘azulfidine ra’ OR azulfin OR benzosulfa OR ‘colo pleon’ OR colo-pleon OR colopleon OR disalazin OR gastropyrin OR ‘pleon ra’ OR ‘pyralin en’ OR rorasul OR rosulfant OR s.a.s.-500 OR salazine OR ‘salazo sulfapyridine’ OR salazodin OR salazopirina OR salazopyridin OR salazopyridine OR salazopyrin OR ‘salazopyrin entabs’ OR salazopyrin-en OR salazopyrina OR salazopyrine OR ‘salazopyrine ec’ OR ‘salazosulfa pyridine’ OR salazosulfpyridine OR ‘salicyl azo sulfapyridine’ OR salicylazosulfapyridin OR salicylazosulfapyridine OR salisulf OR salopyr OR saridine OR ‘sas 500’ OR sulcolon OR sulfasalazine OR sulfasalizine OR sulfosalazine OR sulphasalazine OR zopyrin OR ‘methotrexate’/exp OR methotrexate OR ‘4 amino 10 methylfolic acid’ OR ‘4 amino 10 methylpteroylglutamic acid’ OR ‘4 amino n10 methylpteroylglutamic acid’ OR ‘a methopterine’ OR abitrexate OR amethopterin OR amethopterine OR ametopterine OR antifolan OR biotrexate OR canceren OR ‘cl 14377’ OR cl14377 OR emtexateM OR emthexat OR emthexate OR emtrexate OR enthexate OR farmitrexat OR farmitrexate OR farmotrex OR folex OR ifamet OR imeth OR ‘intradose MTX’ OR jylamvo OR lantarel OR ledertrexate OR maxtrex OR metex OR methoblastin OR methohexate OR methotrate OR methotrexat OR methotrexato OR methoxtrexate OR methrotrexate OR methylaminopterin OR methylaminopterine OR meticil OR metoject OR metothrexate OR metotrexat OR metotrexate OR metotrexin OR metrex OR mexate OR mexate-aq OR ‘mexate-aq preserved’ OR ‘mpi 5004’ OR mpi5004 OR MTX OR neotrexate OR nordimet OR novatrex OR ‘nsc 740’ OR nsc740 OR otrexup OR ‘otrexup pfs’ OR rasuvo OR reumatrex OR rheumatrex OR ‘rheumatrex dose pack’ OR methotrexate OR texate OR texate-t OR texorate OR trexall OR xaken OR xatmep OR zexate OR ‘leflunomide’/exp OR leflunomide OR ‘alpha, alpha, alpha trifluoro 5 methyl 4 isoxazolecarboxy para toluidide’ OR arabloc OR arava OR ‘hwa 486’ OR hwa486 OR repso OR ‘rs 34821’ OR rs34821 OR ‘su 101’ OR su101 OR ‘dapsone’/exp OR dapsone OR ‘4 diaminodiphenylsulfone’ OR ‘4 sulfonyldianiline’ OR ‘4 diaminodiphenyl sulfone’ OR ‘4 diaminodiphenylsulfone’ OR ‘4 sulfonylbisbenzamine’ OR ‘4 sulfonyldianiline’ OR aczone OR atrisone OR avlosulfan OR avlosulfon OR avlosulfone OR ‘4 aminophenyl sulfone’ OR ‘bn 2405’ OR bn2405 OR croysulfone OR dapsoderm-x OR dapson OR ‘dapson-fatol’ OR dapsona OR dds OR ‘diamino diphenyl sulfone’ OR ‘diaminodiphenyl sulfone’ OR diaminodiphenylosulfone OR diaminodiphenylsulfon OR diaminodiphenylsulfone OR diammodiphenylsulfone OR ‘diaphenyl sulfone’ OR diaphenylsulfon OR diaphenylsulfone OR diaphenylsulphone OR diphenason OR diphenasone OR diphone OR disulone OR dopsan OR dumitone OR eporal OR ‘f 1358’ OR f1358 OR lennon-dapsone OR lepravir OR novasulfon OR novophone OR ‘nsc 6091’ OR nsc6091 OR ‘para sulfodianiline’ OR servidapson OR servidapsone OR sulfadione OR sulfadoine OR sulfona OR ‘sulfona mae’ OR ‘sulfone mere’ OR udolac OR ‘glucocorticoid’/exp OR glucocorticoid OR glucocorticoids OR glucocorticoidsteroid OR glucocorticosteroid OR glucocortoid OR glycocorticoid OR glycocorticosteroid OR ‘immunoglobulin’/exp OR immunoglobulin OR ‘antibody protein’ OR endobulin OR ‘flebogamma liquida’ OR gamastan OR ‘gamimmune n’ OR gamimune OR ‘gamma globulin’ OR ‘gamma globulins’ OR ‘gamma immunoglobulin’ OR gamma-globulins OR gammagee OR gammaglobulin OR gammaglobuline OR gammar OR gammimune OR gamulin OR globuman OR ‘glovenin i’ OR Ig OR igam OR igc OR ‘immune gamma globulin’ OR ‘immune globin’ OR ‘immune globulin’ OR ‘immune globuline’ OR ‘immune globulins’ OR ‘immune serum globulin’ OR immuno OR ‘immuno gamma globulin’ OR ‘immuno globulin’ OR immunogammaglobulin OR immunoglobin OR ‘immunoglobulin 17’ OR ‘immunoglobulin c’ OR ‘immunoglobulin c1’ OR ‘immunoglobulin chain’ OR ‘immunoglobulin gamma’ OR ‘immunoglobulin preparation’ OR immunoglobulins OR ‘immunoglobulins, intravenous’ OR immunoprotein OR immunoproteins OR ‘intraglobin f’ OR isiven OR iveegam OR ivega OR ivig OR panglobulin OR sandoglobin OR tegelin OR tegeline OR veinoglobulin OR venoglobulin OR ‘venoglobulin i’ OR ‘venoglobulin-i’

#4 #2 OR #3

#5 #1 AND #4

#6 #5 AND [embase]/lim NOT ([embase]/lim AND [medline]/lim) AND [1-11-2019]/sd

#### Cochrane

#1 MeSH descriptor: [Coronavirus] explode all trees

#2 MeSH descriptor: [Coronaviridae] explode all trees

#3 MeSH descriptor: [Betacoronavirus] explode all trees

#4 MeSH descriptor: [Coronavirus Infections] explode all trees

#5 (COVID 19) OR (COVID-19) OR (2019-nCoV) OR (nCoV) OR (Covid19) OR (SARS-CoV) OR (SARSCov2 or ncov*) OR (SARSCov2) OR (2019 coronavirus*) OR (2019 corona virus*) OR (Coronavirus (COVID-19)) OR (2019 novel coronavirus disease) OR (COVID-19 pandemic) OR (COVID-19 virus infection) OR (coronavirus disease-19) OR (2019 novel coronavirus infection) OR (2019-nCoV infection) OR (coronavirus disease 2019) OR (2019-nCoV disease) OR (COVID-19 virus disease) OR “severe acute respiratory syndrome coronavirus 2” OR (Wuhan coronavirus) OR (Wuhan seafood market pneumonia virus) OR (COVID19 virus) OR (COVID-19 virus) OR (coronavirus disease 2019 virus) OR (SARS-CoV-2) OR (SARS2) OR (2019-nCoV) OR (2019 novel coronavirus)

#6 #1 OR #2 OR #3 OR #4 OR #5

#7 MeSH descriptor: [Interleukin-6] explode all trees

#8 (interleukin 6) OR “IL 6” OR IL-6 OR IL6

#9 Tocilizum* OR altizumab OR actemra OR RHPM-1 OR RG-1569 OR R-1569 OR MSB11456 OR MSB-11456 OR (monoclonal antibody, MRA) OR (RO-4877533) OR roactemra OR anti-IL-6 OR anti-interleukin-6 OR siltuximab OR CLLB8 OR (cClB8 monoclonal antibody) OR Sylvant OR CNTO-328 OR (CNTO 328 monoclonal antibody) OR (monoclonal antibody CNTO328) OR sarilumab OR SAR-153191 OR SAR153191 OR Kevzara OR REGN-88 OR REGN88 OR olokizumab OR CDP-6038 OR CDP6038 OR elsilimomab OR BMS945429 OR ALD518 OR sirukumab OR (CNTO 136) OR CNTO-136 OR CPSI-2364 OR ALX-0061 OR clazakizumab OR ALD-518 OR ALD518 OR BMS-945429 OR sarilumab OR SAR-153191 OR SAR153191 OR Kevzara OR REGN-88 OR REGN88 OR sirukumab OR ARGX-109 OR FE301 OR FM101

#10 MeSH descriptor: [Tumor Necrosis Factors] explode all trees

#11 TNF OR TNF-alpha OR TNF-α OR Anti-TNF

#12 MeSH descriptor: [Tumor Necrosis Factor-alpha] explode all trees

#12 MeSH descriptor: [Infliximab] explode all trees

#13 Infliximab-dyyb OR Remicade OR Renflexis OR Inflectra OR Infliximab-abda OR (Monoclonal Antibody cA2) OR (MAb cA2) OR Infliximab-dyyb

#14 MeSH descriptor: [Etanercept] explode all trees

#15 (TNFR-Fc Fusion Protein) OR (TNR 001) OR (TNT Receptor Fusion Protein) OR TNTR-Fc OR TNR-001 OR TNR001 OR Etanercept-szzs OR Erelzi OR Etanercept-szzs OR (TNFR Fc Fusion Protein)

#16 MeSH descriptor: [Certolizumab Pegol] explode all trees

#17 Certolizumab OR CDP870 OR (CDP 870) OR Cimzia

#18 golimumab OR CNTO-148 OR (CNTO 148) OR Simponi

#19 MeSH descriptor: [Adalimumab] explode all trees

#20 Humira OR Adalimumab-adbm OR Amjevita OR Adalimumab-atto OR Cyltezo OR (D2E7 Antibody)

#21 MeSH descriptor: [Interleukin-1] explode all trees

#22 IL-1 OR IL-1RA OR (IL 1) OR canakinumab OR ilaris OR ACZ-885 OR ACZ885 OR anti-IL-1 OR rilonacept OR ACZ885 OR anakinra

#23 MeSH descriptor: [Interleukin-5] explode all trees

#24 IL-5 OR (IL 5) OR (interleukin 5) OR Anti-IL-5 OR mepolizumab OR Bosatria OR SB-240563 OR SB240563 OR Nucala

#25 MeSH descriptor: [Interleukin-23] explode all trees

#26 “IL-23” OR (Interleukin 23) OR guselkumab OR tildrakizumab OR risankizumab

#27 MeSH descriptor: [Interleukin-17] explode all trees

#28 (Interleukin 17F) OR IL-17F OR brodalumab OR secukinumab OR ixekizumab

#29 MeSH descriptor: [Abatacept] explode all trees

#30 LEA29Y OR BMS224818 OR BMS-224818 OR (BMS 224818) OR Belatacept OR (BMS 188667) OR BMS-188667 OR (CTLA4 Ig Immunoconjugate) OR Nulojix

#31 MeSH descriptor: [Rituximab] explode all trees

#32 (CD20 Antibody) OR (Rituximab CD20 Antibody) OR Mabthera OR (IDEC-C2B8 Antibody) OR (IDEC C2B8 Antibody) OR (IDEC-C2B8) OR (IDEC C2B8) OR GP2013 OR Rituxan OR (CD20 Antigen) OR (CD20 Antigens)

#33 belimumab OR (BEL-114333) OR BEL114333 OR HGS-1006 OR HGS1006 OR LymphoStat-B OR GSK-1550188 OR GSK1550188 OR Benlysta

#34 IL-17A OR IL-17 OR (IL 17)

#35 MeSH descriptor: [Interleukin-12] explode all trees

#36 MeSH descriptor: [Interleukin-23] explode all trees

#37 IL-23 OR (IL 23) OR (interleukin 23)

#38 MeSH descriptor: [Ustekinumab] explode all trees

#39 Stelara OR (CNTO 1275) OR CNTO-1275

#40 briakinumab OR A-796874.0 OR BSF-415977 OR (BSF 415977) OR WAY-165772 OR LU-415977 OR (LU 415977) OR J-695 OR J695 OR ABT-874 OR (ABT-874 antibody, human) OR Anti-C5 OR eculizumab OR Alexion OR Soliris OR H5G1.1 OR H5G1-1 OR H5G1

#41 Apremilast OR Otezla OR Tasocitinib OR (tofacitinib citrate) OR Xeljanz OR Baricitinib OR Olumiant

#42 Azathioprine OR Azothioprine OR Imurel OR Imuran OR Immuran

#43 MeSH descriptor: [Mycophenolic Acid] explode all trees

#44 (Mycophenolate Mofetil) OR (Mycophenolate Sodium) OR Myfortic

#45 MeSH descriptor: [Cyclophosphamide] explode all trees

#46 Sendoxan OR Cytophosphan OR Procytox OR Cyclophosphane OR Neosar OR Cytoxan OR Cytophosphane

#47 MeSH descriptor: [Cyclosporins] in all MeSH products

#48 (CsA Neoral) OR CsANeoral OR CsA-Neoral OR Neoral OR CyA-NOFM OR CyA NOF OR Cyclosporin OR Ciclosporin OR “Cyclosporine A” OR Sandimmune OR Sandimmun

#49 MeSH descriptor: [Tacrolimus] explode all trees

#50 Prograf OR Prograft

#51 MeSH descriptor: [Chloroquine] explode all trees

#52 Nivaquine OR Aralen OR Arechine OR Arequin OR Chlorochin OR Chingamin OR Khingamin

#53 MeSH descriptor: [Hydroxychloroquine] explode all trees

#54 Plaquenil OR Hydroxychlorochin OR Oxychlorochin OR Oxychloroquine

#55 MeSH descriptor: [Sulfasalazine] explode all trees

#56 Salicylazosulfapyridine OR Sulphasalazine OR Salazosulfapyridine OR (Colo Pleon) OR Pleon OR Colo-Pleon OR Azulfadine OR Azulfidine OR Asulfidine OR Sulfasalazin-Heyl OR Sulfasalazin OR Salazopyrin OR Ulcol OR Ucine OR “Pyralin EN”

#57 MeSH descriptor: [Methotrexate] explode all trees

#58 Methotrexate OR Mexate OR Amethopterin

#59 MeSH descriptor: [Leflunomide] explode all trees

#60 “N-(4-Trifluoromethyphenyl)-5-methylisoxazole-4-carboxamide” OR Arava OR (SU101) OR (HWA 486) OR HWA486 OR HWA-486

#61 MeSH descriptor: [Dapsone] explode all trees

#62 Sulfona OR “4,4'-Diaminophenyl Sulfone” OR Diaphenylsulfone OR DADPS OR “4,4' Diaminophenyl Sulfone” OR “Sulfone, 4,4'-Diaminophenyl” OR Diaminodiphenylsulfone OR Sulfonyldianiline OR Avlosulfone OR Disulone OR “Dapsoderm-X” OR “Dapson-Fatol”

#63 MeSH descriptor: [Glucocorticoids] explode all trees

#64 “Glucocorticoid Effect” OR Glucocorticoid

#65 MeSH descriptor: [Immunoglobulins] explode all trees

#66 Immunoglobulin OR Globulins

### SCOPUS

#1 TITLE-ABS-KEY(coronavirus)

#2 TITLE-ABS-KEY(coronaviridae)

#3 TITLE-ABS-KEY(“Coronavirus Infections”)

#4 TITLE-ABS-KEY(betacoronavirus)

#5 (COVID 19) OR (COVID-19) OR (2019-nCoV) OR (nCoV) OR (Covid19) OR (SARS-CoV) OR (SARSCov2 or ncov*) OR (SARSCov2) OR (2019 coronavirus*) OR (2019 corona virus*) OR (Coronavirus (COVID-19)) OR (2019 novel coronavirus disease) OR (COVID-19 pandemic) OR (COVID-19 virus infection) OR (coronavirus disease-19) OR (2019 novel coronavirus infection) OR (2019-nCoV infection) OR (coronavirus disease 2019) OR (2019-nCoV disease) OR (COVID-19 virus disease) OR (severe acute respiratory syndrome coronavirus 2) OR (Wuhan coronavirus) OR (Wuhan seafood market pneumonia virus) OR (COVID19 virus) OR (COVID-19 virus) OR (coronavirus disease 2019 virus) OR (SARS-CoV-2) OR (SARS2) OR (2019-nCoV) OR (2019 novel coronavirus)

#6 #1 OR #2 OR #3 OR #4 OR #5

#7 TITLE-ABS-KEY(Interleukin-6)

#8 TITLE-ABS-KEY(“Tumor Necrosis Factors”)

#9 TITLE-ABS-KEY(“Tumor Necrosis Factor-alpha”)

#10 TITLE-ABS-KEY(Infliximab)

#11 TITLE-ABS-KEY(Etanercept)

#12 TITLE-ABS-KEY(“Certolizumab Pegol”)

#13 TITLE-ABS-KEY(Adalimumab)

#14 TITLE-ABS-KEY(Interleukin-1)

#15 TITLE-ABS-KEY(Interleukin-5)

#16 TITLE-ABS-KEY(Interleukin-23)

#17 TITLE-ABS-KEY(Interleukin-17)

#18 TITLE-ABS-KEY(Abatacept)

#19 TITLE-ABS-KEY(Rituximab)

#20 TITLE-ABS-KEY(Interleukin-12)

#21 TITLE-ABS-KEY(Interleukin-23)

#22 TITLE-ABS-KEY(Ustekinumab)

#23 TITLE-ABS-KEY(“Mycophenolic Acid”)

#24 TITLE-ABS-KEY(Cyclophosphamide)

#25 TITLE-ABS-KEY(Cyclosporins)

#26 TITLE-ABS-KEY(Tacrolimus)

#27 TITLE-ABS-KEY(Chloroquine)

#28 TITLE-ABS-KEY(Hydroxychloroquine)

#29 TITLE-ABS-KEY(Sulfasalazine)

#30 TITLE-ABS-KEY(Methotrexate)

#31 TITLE-ABS-KEY(Leflunomide)

#32 TITLE-ABS-KEY(Dapsone)

#33 TITLE-ABS-KEY(Glucocorticoids)

#34 TITLE-ABS-KEY(Immunoglobulins)

#35 (interleukin 6) OR “IL 6” OR IL-6 OR IL6 OR Tocilizum* OR altizumab OR actemra OR RHPM-1 OR RG-1569 OR R-1569 OR MSB11456 OR MSB-11456 OR (monoclonal antibody, MRA) OR (RO-4877533) OR roactemra OR anti-IL-6 OR anti-interleukin-6 OR siltuximab OR CLLB8 OR (cClB8 monoclonal antibody) OR Sylvant OR CNTO-328 OR (CNTO 328 monoclonal antibody) OR (monoclonal antibody CNTO328) OR sarilumab OR SAR-153191 OR SAR153191 OR Kevzara OR REGN-88 OR REGN88 OR olokizumab OR CDP-6038 OR CDP6038 OR elsilimomab OR BMS945429 OR ALD518 OR sirukumab OR (CNTO 136) OR CNTO-136 OR CPSI-2364 OR ALX-0061 OR clazakizumab OR ALD-518 OR ALD518 OR BMS-945429 OR sarilumab OR SAR-153191 OR SAR153191 OR Kevzara OR REGN-88 OR REGN88 OR sirukumab OR ARGX-109 OR FE301 OR FM101 OR TNF OR TNF-alpha OR TNF-α OR Anti-TNF OR Infliximab-dyyb OR Remicade OR Renflexis OR Inflectra OR Infliximab-abda OR (Monoclonal Antibody cA2) OR (MAb cA2) OR Infliximab-dyyb OR (TNFR-Fc Fusion Protein) OR (TNR 001) OR (TNT Receptor Fusion Protein) OR TNTR-Fc OR TNR-001 OR TNR001 OR Etanercept-szzs OR Erelzi OR Etanercept-szzs OR (TNFR Fc Fusion Protein) OR Certolizumab Pegol OR Certolizumab OR CDP870 OR (CDP 870) OR Cimzia OR golimumab OR CNTO-148 OR (CNTO 148) OR Simponi OR Humira OR Adalimumab-adbm OR Amjevita OR Adalimumab-atto OR Cyltezo OR (D2E7 Antibody) OR IL-1 OR IL-1RA OR (IL 1) OR canakinumab OR ilaris OR ACZ-885 OR ACZ885 OR anti-IL-1 OR rilonacept OR ACZ885 OR anakinra OR IL-5 OR (IL 5) OR (interleukin 5) OR Anti-IL-5 OR mepolizumab OR Bosatria OR SB-240563 OR SB240563 OR Nucala OR “IL-23” OR (Interleukin 23) OR guselkumab OR tildrakizumab OR risankizumab OR (Interleukin 17F) OR IL-17F OR brodalumab OR secukinumab OR ixekizumab OR LEA29Y OR BMS224818 OR BMS-224818 OR (BMS 224818) OR Belatacept OR (BMS 188667) OR BMS-188667 OR (CTLA4 Ig Immunoconjugate) OR Nulojix OR (CD20 Antibody) OR (Rituximab CD20 Antibody) OR Mabthera OR (IDEC-C2B8 Antibody) OR (IDEC C2B8 Antibody) OR (IDEC-C2B8) OR (IDEC C2B8) OR GP2013 OR Rituxan OR (CD20 Antigen) OR (CD20 Antigens) OR belimumab OR (BEL-114333) OR BEL114333 OR HGS-1006 OR HGS1006 OR LymphoStat-B OR GSK-1550188 OR GSK1550188 OR Benlysta OR IL-17A OR IL-17 OR (IL 17) OR IL-23 OR (IL 23) OR (interleukin 23) OR Stelara OR (CNTO 1275) OR CNTO-1275 OR briakinumab OR A-796874.0 OR BSF-415977 OR (BSF 415977) OR WAY-165772 OR LU-415977 OR (LU 415977) OR J-695 OR J695 OR ABT-874 OR (ABT-874 antibody, human) OR Anti-C5 OR eculizumab OR Alexion OR Soliris OR H5G1.1 OR H5G1-1 OR H5G1 OR Apremilast OR Otezla OR Tasocitinib OR (tofacitinib citrate) OR Xeljanz OR Baricitinib OR Olumiant OR Azathioprine OR Azothioprine OR Imurel OR Imuran OR Immuran OR (Mycophenolate Mofetil) OR (Mycophenolate Sodium) OR Myfortic OR Sendoxan OR Cytophosphan OR Procytox OR Cyclophosphane OR Neosar OR Cytoxan OR Cytophosphane OR (CsA Neoral) OR CsANeoral OR CsA-Neoral OR Neoral OR CyA-NOFM OR CyA NOF OR Cyclosporin OR Ciclosporin OR “Cyclosporine A” OR Sandimmune OR Sandimmun OR Prograf OR Prograft OR Nivaquine OR Aralen OR Arechine OR Arequin OR Chlorochin OR Chingamin OR Khingamin OR Plaquenil OR Hydroxychlorochin OR Oxychlorochin OR Oxychloroquine OR Salicylazosulfapyridine OR Sulphasalazine OR Salazosulfapyridine OR (Colo Pleon) OR Pleon OR Colo-Pleon OR Azulfadine OR Azulfidine OR Asulfidine OR Sulfasalazin-Heyl OR Sulfasalazin OR Salazopyrin OR Ulcol OR Ucine OR “Pyralin EN” OR Methotrexate OR Mexate OR Amethopterin OR “N-(4-Trifluoromethyphenyl)-5-methylisoxazole-4-carboxamide” OR Arava OR (SU101) OR (HWA 486) OR HWA486 OR HWA-486 OR Sulfona OR “4,4'-Diaminophenyl Sulfone” OR Diaphenylsulfone OR DADPS OR “4,4' Diaminophenyl Sulfone” OR “Sulfone, 4,4'-Diaminophenyl” OR Diaminodiphenylsulfone OR Sulfonyldianiline OR Avlosulfone OR Disulone OR “Dapsoderm-X” OR “Dapson-Fatol” OR “Glucocorticoid Effect” OR Glucocorticoid OR Immunoglobulin OR Globulins

#36 #7 OR #8 OR #9 #10 OR #11 OR #12 OR #13 OR #14 OR #15 OR #16 OR #17 OR #18 OR #19 OR #20 OR #21 OR #22 OR #23 OR #24 OR #25 OR #26 OR #27 OR #28 OR #29 #30 OR #31 OR #32 OR #33 OR #34 OR #35

#37 #6 AND #36

#38 #37 AND (LIMIT-TO(PUBYEAR, 2020) OR LIMIT-TO(PUBYEAR, 2019))

### WEB OF SCIENCE

TS=(COVID 19 OR COVID-19 OR 2019-nCoV OR nCoV OR Covid19 OR SARS-CoV OR SARSCov2 or ncov* OR 2019 coronavirus* OR 2019 corona virus* OR Coronavirus OR 2019 novel coronavirus disease OR COVID-19 pandemic OR COVID-19 virus infection OR coronavirus disease-19 OR 2019 novel coronavirus infection OR 2019-nCoV infection OR coronavirus disease 2019 OR 2019-nCoV disease OR COVID-19 virus disease OR severe acute respiratory syndrome coronavirus 2 OR Wuhan coronavirus OR Wuhan seafood market pneumonia virus OR COVID19 virus OR COVID-19 virus OR coronavirus disease 2019 virus OR SARS-CoV-2 OR SARS2 OR 2019 novel coronavirus) AND TS=(Interleukin-6 OR interleukin 6 OR IL 6 OR IL-6 OR IL6 OR Tocilizum* OR altizumab OR actemra OR RHPM-1 OR RG-1569 OR R-1569 OR MSB11456 OR MSB-11456 OR monoclonal antibody, MRA OR RO-4877533 OR roactemra OR anti-IL-6 OR anti-interleukin-6 OR siltuximab OR CLLB8 OR cClB8 monoclonal antibody OR Sylvant OR CNTO-328 OR CNTO 328 monoclonal antibody OR monoclonal antibody CNTO328 OR sarilumab OR SAR-153191 OR SAR153191 OR Kevzara OR REGN-88 OR REGN88 OR olokizumab OR CDP-6038 OR CDP6038 OR elsilimomab OR BMS945429 OR ALD518 OR sirukumab OR CNTO 136 OR CNTO-136 OR CPSI-2364 OR ALX-0061 OR clazakizumab OR ALD-518 OR ALD518 OR BMS-945429 OR sarilumab OR SAR-153191 OR SAR153191 OR Kevzara OR REGN-88 OR REGN88 OR sirukumab OR ARGX-109 OR FE301 OR FM101 OR Tumor Necrosis Factors OR Tumor Necrosis Factor-alpha OR TNF OR TNF-alpha OR TNF-α OR Anti-TNF OR Infliximab-dyyb OR Remicade OR Renflexis OR Inflectra OR Infliximab OR Infliximab-abda OR Monoclonal Antibody cA2 OR MAb cA2 OR Infliximab-dyyb OR TNFR-Fc Fusion Protein OR TNR 001 OR TNT Receptor Fusion Protein OR TNTR-Fc OR TNR-001 OR TNR001 OR Etanercept OR Etanercept-szzs OR Erelzi OR Etanercept-szzs OR TNFR Fc Fusion Protein OR Certolizumab OR CDP870 OR CDP 870 OR Cimzia OR golimumab OR CNTO-148 OR CNTO 148 OR Simponi OR Humira OR Adalimumab OR Adalimumab-adbm OR Amjevita OR Adalimumab-atto OR Cyltezo OR D2E7 Antibody OR Interleukin-1 OR IL-1 OR IL-1RA OR IL 1 OR canakinumab OR ilaris OR ACZ-885 OR ACZ885 OR anti-IL-1 OR rilonacept OR ACZ885 OR anakinra OR Interleukin-5 OR IL-5 OR IL 5 OR interleukin 5 OR Anti-IL-5 OR mepolizumab OR Bosatria OR SB-240563 OR SB240563 OR Nucala OR Interleukin-23 OR IL-23 OR Interleukin 23 OR guselkumab OR tildrakizumab OR risankizumab OR Interleukin-17 OR Interleukin 17F OR IL-17F OR brodalumab OR secukinumab OR ixekizumab OR abatacept OR LEA29Y OR BMS224818 OR BMS-224818 OR BMS 224818 OR Belatacept OR BMS 188667 OR BMS-188667 OR CTLA4 Ig Immunoconjugate OR Nulojix OR CD20 Antibody OR Rituximab OR Rituximab CD20 Antibody OR Mabthera OR IDEC-C2B8 Antibody OR IDEC C2B8 Antibody OR IDEC-C2B8 OR IDEC C2B8 OR GP2013 OR Rituxan OR CD20 Antigen OR CD20 Antigens OR belimumab OR BEL-114333 OR BEL114333 OR HGS-1006 OR HGS1006 OR LymphoStat-B OR GSK-1550188 OR GSK1550188 OR Benlysta OR IL-17A OR IL-17 OR IL 17 OR IL-23 OR Interleukin-12 OR Interleukin-23 OR IL 23 OR interleukin 23 OR Stelara OR CNTO 1275 OR CNTO-1275 OR ustekinumab OR briakinumab OR A-796874.0 OR BSF-415977 OR BSF 415977 OR WAY-165772 OR LU-415977 OR LU 415977 OR J-695 OR J695 OR ABT-874 OR ABT-874 antibody, human OR Anti-C5 OR eculizumab OR Alexion OR Soliris OR H5G1.1 OR H5G1-1 OR H5G1 OR Apremilast OR Otezla OR Tasocitinib OR tofacitinib citrate OR Xeljanz OR Baricitinib OR Olumiant OR Azathioprine OR Azothioprine OR Imurel OR Imuran OR Immuran OR Mycophenolic Acid OR Mycophenolate Mofetil OR Mycophenolate Sodium OR Myfortic OR Sendoxan OR Cyclophosphamide OR Cytophosphan OR Procytox OR Cyclophosphane OR Neosar OR Cytoxan OR Cytophosphane OR CsA Neoral OR CsANeoral OR CsA-Neoral OR Neoral OR CyA-NOFM OR CyA NOF OR Cyclosporins OR Cyclosporin OR Ciclosporin OR “Cyclosporine A” OR Sandimmune OR Sandimmun OR Tacrolimus OR Prograf OR Prograft OR Nivaquine OR Aralen OR Arechine OR Arequin OR Chloroquine OR Chlorochin OR Chingamin OR Khingamin OR Plaquenil OR Hydroxychloroquine OR Hydroxychlorochin OR Oxychlorochin OR Oxychloroquine OR Salicylazosulfapyridine OR Sulfasalazine OR Sulphasalazine OR Salazosulfapyridine OR Colo Pleon OR Pleon OR Colo-Pleon OR Azulfadine OR Azulfidine OR Asulfidine OR Sulfasalazin-Heyl OR Sulfasalazin OR Salazopyrin OR Ulcol OR Ucine OR Pyralin EN OR Methotrexate OR Mexate OR Amethopterin OR N-(4-Trifluoromethyphenyl)-5-methylisoxazole-4-carboxamide OR Arava OR SU101 OR HWA 486 OR HWA486 OR HWA-486 OR Sulfona OR 4,4'-Diaminophenyl Sulfone OR Diaphenylsulfone OR DADPS OR 4,4' Diaminophenyl Sulfone OR Sulfone, 4,4'-Diaminophenyl OR Diaminodiphenylsulfone OR Leflunomide OR Sulfonyldianiline OR Avlosulfone OR Disulone OR Dapsone OR Dapsoderm-X OR Dapson-Fatol OR Glucocorticoids OR Glucocorticoid Effect OR Glucocorticoid OR Immunoglobulins OR Immunoglobulin OR Globulins)

#### BVS regional portal – LILACS

MH:”Infecções por Coronavirus” OR (Infecções por Coronavirus) OR (Infecciones por Coronavirus) OR (Coronavirus Infections) OR (COVID-19) OR (COVID 19) OR (Doença pelo Novo Coronavírus (2019-nCoV)) OR (Doença por Coronavírus 2019-nCoV) OR (Doença por Novo Coronavírus (2019-nCoV)) OR (Epidemia de Pneumonia por Coronavirus de Wuhan) OR (Epidemia de Pneumonia por Coronavírus de Wuhan) OR (Epidemia de Pneumonia por Coronavírus de Wuhan de 2019-2020) OR (Epidemia de Pneumonia por Coronavírus em Wuhan) OR (Epidemia de Pneumonia por Coronavírus em Wuhan de 2019-2020) OR (Epidemia de Pneumonia por Novo Coronavírus de 2019-2020) OR (Epidemia pelo Coronavírus de Wuhan) OR (Epidemia pelo Coronavírus em Wuhan) OR (Epidemia pelo Novo Coronavírus (2019-nCoV)) OR (Epidemia pelo Novo Coronavírus 2019) OR (Epidemia por 2019-nCoV) OR (Epidemia por Coronavírus de Wuhan) OR (Epidemia por Coronavírus em Wuhan) OR (Epidemia por Novo Coronavírus (2019-nCoV)) OR (Epidemia por Novo Coronavírus 2019) OR (Febre de Pneumonia por Coronavírus de Wuhan) OR (Infecção pelo Coronavírus 2019-nCoV) OR (Infecção pelo Coronavírus de Wuhan) OR (Infecção por Coronavirus 2019-nCoV) OR (Infecção por Coronavírus 2019-nCoV) OR (Infecção por Coronavírus de Wuhan) OR (Infecções por Coronavírus) OR (Pneumonia do Mercado de Frutos do Mar de Wuhan) OR (Pneumonia no Mercado de Frutos do Mar de Wuhan) OR (Pneumonia por Coronavírus de Wuhan) OR (Pneumonia por Novo Coronavírus de 2019-2020) OR (Surto de Coronavírus de Wuhan) OR (Surto de Pneumonia da China 2019-2020) OR (Surto de Pneumonia na China 2019-2020) OR (Surto pelo Coronavírus 2019-nCoV) OR (Surto pelo Coronavírus de Wuhan) OR (Surto pelo Coronavírus de Wuhan de 2019-2020) OR (Surto pelo Novo Coronavírus (2019-nCoV)) OR (Surto pelo Novo Coronavírus 2019) OR (Surto por 2019-nCoV) OR (Surto por Coronavírus 2019-nCoV) OR (Surto por Coronavírus de Wuhan) OR (Surto por Coronavírus de Wuhan de 2019-2020) OR (Surto por Novo Coronavírus (2019-nCoV)) OR (Surto por Novo Coronavírus 2019) OR (Síndrome Respiratória do Oriente Médio) OR (Síndrome Respiratória do Oriente Médio (MERS)) OR (Síndrome Respiratória do Oriente Médio (MERS-CoV)) OR (Síndrome Respiratória do Oriente Médio por Coronavírus) OR MH:C01.925.782.600.550.200$

Filter: Publication year: 2019-2020
